# Urinary vitamin D-binding protein as a marker of ovarian reserve

**DOI:** 10.1186/s12958-021-00762-9

**Published:** 2021-06-01

**Authors:** Sanglin Li, Lina Hu, Chanyu Zhang

**Affiliations:** Reproductive Medicine Center, Department of Gynecology and Obstetrics, The Second Affiliated Hospital, Chongqing Medical University, 76 Linjiang Road, Chongqing, 400010 China

**Keywords:** Vitamin D-binding protein, Diminished ovarian reserve, Polycystic ovary syndrome, Proteomics, iTRAQ

## Abstract

**Background:**

Ovarian reserve reflects the quality and quantity of available oocytes and has become an indispensable measure for the better understanding of reproductive potential. Proteomic approaches are especially helpful in discerning differential protein expression patterns associated with normal and diseased states and, thus, proteomic analyses are increasingly used to identify clinically useful biomarkers. The aim of this study was to investigate proteins secreted in the urine of patients with different ovarian reserve by proteomic techniques to identify potential markers for assessing ovarian reserve.

**Methods:**

Urine samples were obtained from patients with polycystic ovary syndrome (PCOS) and diminished ovarian reserve (DOR), and from normal control (NC)participants. We used isobaric tags for relative and absolute quantification (iTRAQ) technology combined with mass spectrometry analysis to identify candidate urinary proteins in the three groups. The selected proteins were confirmed using western blot analysis and enzyme-linked immunosorbent assay (ELISA). Diagnostic performance of the selected proteins was assessed using receiver operating characteristic analysis.

**Results:**

When Compared with NC samples, 285 differentially expressed proteins (DEPs) were identified in the DOR samples and 372 in the PCOS samples. By analyzing the intersection of the two groups of DEPs, we found 26 proteins with different expression trends in the DOR and PCOS groups. Vitamin D-binding protein (VDBP) was the key protein for the protein-protein interaction network. ELISA quantification of urinary VDBP revealed the highest levels in the PCOS group, followed by the NC group and the lowest levels in the DOR group (115.90 ± 26.02, 81.86 ± 23.92 and 52.84 ± 21.37 ng/ml, respectively; *P* < 0.05). As a diagnostic marker, VDBP had a sensitivity of 67.4% and a specificity of 91.8% for DOR, and a sensitivity of 93.8% and a specificity of 77.6% for PCOS.

**Conclusions:**

Urinary VDBP is closely associated with ovarian reserve and can be considered as a novel noninvasive biomarker of ovarian reserve. However, studies including large sample sizes are needed to validate these results.

## Introduction

Ovarian reserve is a concept that reflects the quality and quantity of ovarian follicles at a given point in time and, therefore, predicts potential ovarian function [1]. Diminished ovarian reserve (DOR), a decreased follicle pool because of a reduction in growing follicles and diminished oocyte quality, is a troubling issue in reproductive medicine. DOR is often associated with poor ovarian response (POR), a high cancellation rate, and a significant decline in pregnancy rates during in vitro fertilization (IVF) cycles [2]. On the contrary, as one of the most common reproductive endocrine disorders in women, polycystic ovary syndrome (PCOS) is characterized by polycystic changes of the ovary. PCOS is usually associated with a hyper-response to ovarian stimulation for assisted reproduction techniques. Therefore, PCOS can be considered as high ovarian reserve. Evaluation of ovarian reserve can identify patients who may experience poor response or hyper-response to exogenous gonadotrophins and can aid in the personalization of treatment to achieve good responses and minimize risks for assisted reproduction techniques [3].

Several predictors of ovarian reserve have been identified, including patient age, basal levels of follicle-stimulating hormone (FSH), anti-mullerian hormone (AMH), and antral follicle count (AFC). Ovarian reserve is age-related, but ovarian reserve may vary in women of the same chronological age [4]. FSH, AFC, or AMH serum levels are the most frequently used criteria for assessing ovarian reserve. Furthermore, most of these measures have limited predictive value. For example, basal FSH fluctuates during the menstrual cycle. AFC maybe vary depending on instrumentation and operator variability and the failure to establish category defining criteria. AMH is generally considered to be non-cyclic throughout normal menstrual cycles and of good predictive value [5, 6]. However, a recent study found that serum AMH levels were significantly lower in the late luteal phase, compared with the early follicular phase, with a pattern similar to pituitary FSH [7]. Furthermore, there is no uniform standard for these predictors in evaluating ovarian reserve [8].

The value of evaluating ovarian reserve with a single predictor is limited, and it is advisable to use a combination of predictors. As such, the search for new biomarkers for ovarian reserve has attracted much attention.

Proteomic approaches in healthy and pathological samples are especially helpful to facilitate discerning differential protein expression patterns associated with normal and diseased states. Proteomic analyses are increasingly used to identify clinically useful biomarkers and can assist in diagnosis and disease staging [9–11]. Most of the biomarkers used in clinical evaluations are found in blood, but few studies have sought ovarian reserve biomarkers in urine. In the present study, we aimed to discover urinary diagnostic biomarkers of ovarian reserve based on proteomic technology and evaluate the diagnostic value of the identified proteins in a validation cohort.

## Materials and methods

### Subjects and urine collection

For the discovery phase, urine specimens were obtained from patients with polycystic ovary syndrome (PCOS; *n* = 10), diminished ovarian reserve (DOR; *n* = 10), and normal control participants (NC; *n* = 10) in the Reproductive Medicine Center of the Second Affiliated Hospital of Chongqing Medical University (Chongqing, China). For the validation phase, 140 urine specimens of participants were obtained, including 43 DOR, 49 NC, and 48 PCOS patients, which were entirely separated from the discovery cohort samples. This study was approved by the Ethics Committee of the Chongqing Medical University. All subjects signed informed consent forms prior to their inclusion in this study. DOR was defined as a basal FSH level greater than 10 mIU/mL or AFC less than 5 [12]. The diagnosis of PCOS was performed as described in the literature [13]. Excluding PCOS or DOR,or other endocrine disorders,Participants with regular menstruation and normal ovulation were considered as normal controls. All participants were ≤35 years of age and had no history of endometriosis,or ovarian tumor,and other endocrine disease (thyroid disorders,hyperprolactinemia, Cushing syndrome,etc). They did not take oral contraceptives,any other hormonal or steroid drug and vitamin D supplements in the past 3 months.

Each urine sample was collected in the early follicular phase (days 2 to 4) of the menstrual cycle. Urine samples were immediately centrifuged at 1000 g for 10 min and sediment-free urine samples were obtained. Urine aliquots were frozen at − 80 °C until used for further analysis.

Serum samples were collected along with urine samples from the participants.

Serum samples were analyzed for FSH,AMH and 25-hydroxy vitamin D [25(OH)D]. All samples were measured using commercial assays according to the manufacturer’s protocols. Serum FSH was measured using an Elecsys FSH electrochemiluminescence immunoassay kit (Roche Diagnostics GmbH,Mannheim, Germany) with immunoassay device (Cobas e601) and presented in mIU/mL.Serum AMH was measured using an Access AMH chemiluminescence immunoassay kit (Immunotech S.A.S. a Beckman Coulter Company,13,276 Marseille Cedex 9,France) with immunoassay device(Acess 2) and presented in ng/mL.Serum 25(OH) D was measured using a LIAISON 25 OH vitamin D TOTAL Assay kit (DiaSorin Inc., Stillwater, Minnesota 55,082,USA) with immunoassay device (LIAISON XL) and presented in nmol/L.The clinical characteristics of the participants, including age, body mass index (BMI), FSH levels, AFC,AMH levels,and 25(OH) D levels, are shown in Table 1.

**Table 1:** Clinical characteristics of patients enrolled in the study

Characteristic	DOR (*n = 53*)	NC (*n = 59*)	PCOS (*n =* 58)	*P*
Age, yr	29.45 ± 3.13	28.85 ± 2.89	28.31 ± 2.89	0.132
BMI (kg/m^2)^	20.98 ± 2.06	21.40 ± 1.79	22.31 ± 2.38	< 0.01
FSH (mIU/ml)	11.11 ± 4.60	6.76 ± 1.95	6.97 ± 1.26	< 0.01
AFC	4.06 ± 2.03	11.97 ± 3.48	22.12 ± 2.90	< 0.01
AMH (ng/ml)	1.10 ± 0.71	3.34 ± 1.05	8.27 ± 3.43	< 0.01
25OH-D (nmol/l)	38.00 ± 12.25	38.23 ± 12.37	38.11 ± 12.82	0.995

Data are expressed as mean ± SD

The number of cases in each group is the sum of the number of cases in the discovery phase and the validation phase

### Urinary protein extraction and isobaric tags for relative and absolute quantification (iTRAQ) labeling

**Fig. 1:**
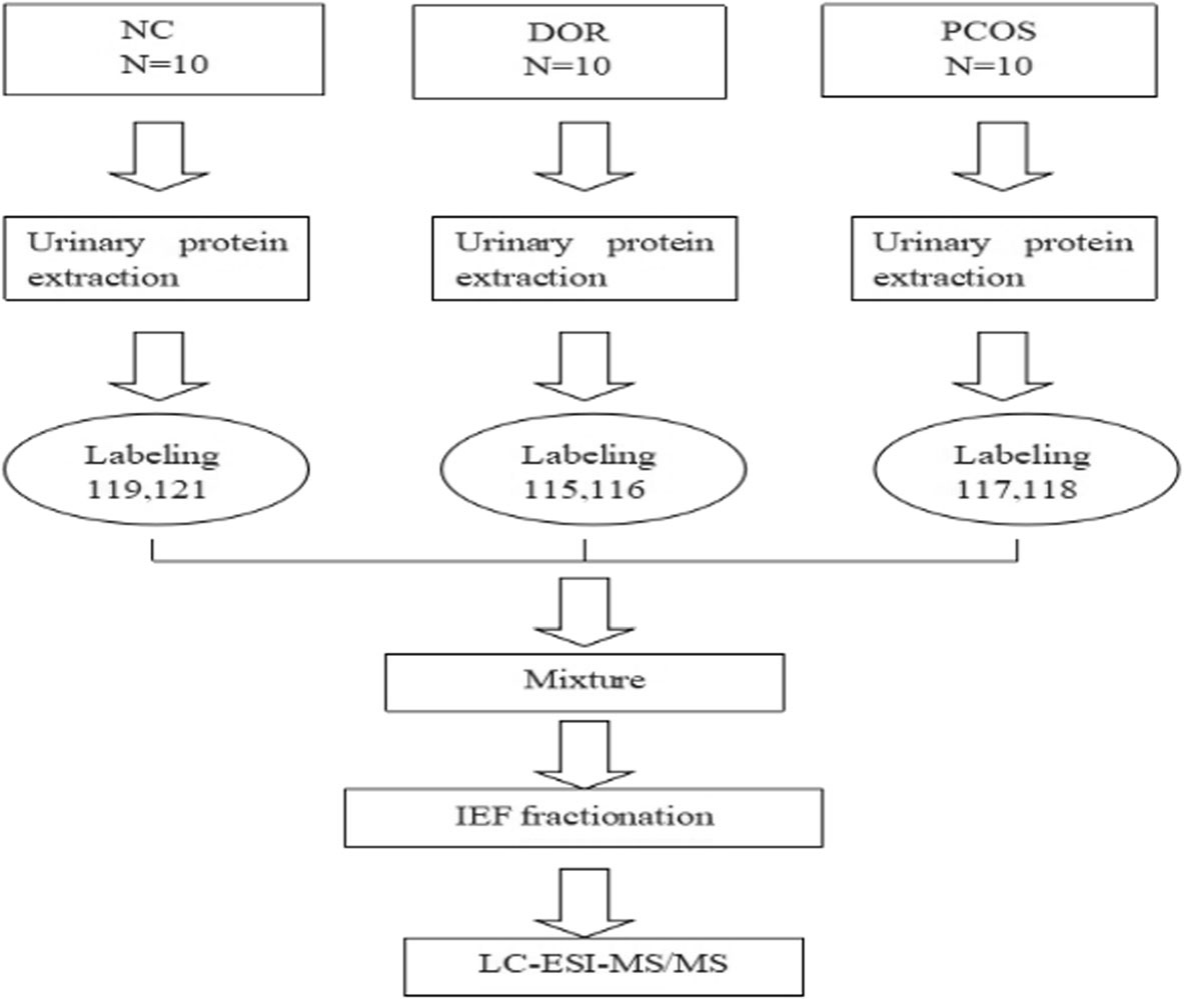
The iTRAQ-based mass spectrometry workflow

The urine samples were centrifuged at 1000 g for 10 min to pellet cellular debris. The supernatants were ultra-filtered with Amicon Ultra-15 centrifugal filter devices (3 K for discovery phase, 30 K for validation phase) at 3320 g for 45 min, and ~ 200 μl concentrated urine was obtained from each 15-ml supernatant sample. Urinary proteins were precipitated with precooled acetone and dissolved again in dissolution buffer, denatured, cysteine-blocked, digested with 2 μg of sequencing grade-modified trypsin, and labeled using the iTRAQ reagents (NC, 119 tag; DOR, 115 tag; PCOS, 117 tag). For reliable results, duplicate labeling was performed for each sample set with iTRAQ tags 121 for NC, 116 for DOR, and 118 for PCOS samples. The labeled samples were pooled before further analysis. Mass spectrometry was carried out with liquid chromatography on a Triple TOF 5600 system. The discovery phase workflow, which included urine specimens derived from DOR, PCOS, and NC individuals that were analyzed via liquid chromatography-electrospray ionization-tandem mass spectrometry (LC-ESI-MS/MS) analysis is detailed in Fig. 1.

### Construction of protein-protein interaction network and key protein analysis

To analyze the connections among the differentially expressed proteins (DEPs), DEPs were uploaded to Search Tool for the Retrieval of Interacting Genes (STRING; https://string-db.org/), a database of known and predicted protein-protein interactions, and the results with a minimum interaction score of 0.4 were visualized in Cytoscape. Furthermore, CytoHubba, a Cytoscape plugin application providing a user-friendly interface to explore important nodes in biological networks, was used with the maximal clique centrality (MCC) method to explore the protein-protein interaction (PPI) network for key proteins.

### Western blot analysis

To validate the results of the proteomic analysis of the urine samples, the same sets of urine samples (discovery cohort) were subjected to immunoblot analysis. Each sample was ultra-filtered with 30 K Amicon Ultra-15 centrifugal filter devices. Approximately 200 μl concentrated protein solution was obtained, added to 5 loading buffer (Beyotime, China) and boiled for 5 min. Proteins were separated via SDS-PAGE and transferred onto PVDF membranes (Millipore, Bedford, MA, USA). The membranes were blocked and incubated with anti-vitamin D binding protein (VDBP) antibody (Abcam, Cambridge, MA, USA) overnight at 4 °C. The membranes were washed with TBS-Tween and incubated with secondary antibodies (1:1000) for 1.5 h at room temperature. Anti-GAPDH (1:1000; Cell Signaling Technology, Danvers, MA, USA) was used as an internal control in the cell experiments. Protein bands were visualized using enhanced chemiluminescence equipment (Pierce Chemical, Dallas, TX, USA).

### Enzyme-linked immunosorbent assay (ELISA)

Levels of VDBP in the urine samples were determined in all 140 validation cohort samples with commercially available human VDBP ELISA kits (R&D Systems, Mineapolis, MN, USA), according to the manufacturer’s protocols. The minimum detectable level of VDBP was determined to be 0.338 ng/ml. All measurements were carried out in duplicate. To correct for dilution variability, creatinine was measured in all urine specimens, and the urine VDBP results were normalized for creatinine concentration by dividing the VDBP concentrations by creatinine concentrations.

### Statistical analysis

Statistical analyses were performed using SPSS 20.0 (IBM, Armonk, NY, USA). Data were expressed as mean + SD and were tested by ANOVA test and Student-Newman-Keuls (SNK) test. Pearson correlation was used to calculate the magnitude and direction of the correlations between measured variables. Diagnostic performance of the selected proteins was assessed using receiver operating characteristic (ROC) curve analysis. The diagnostic utility of the test can be expressed as the area under the ROC curve (AUC), which was calculated as a measure of the ability of each potential biomarker to discriminate between patient and control cases. An AUC of 0.5 indicates classification assigned by chance. Based on ROC analysis, the best statistical cut-off value of VDBP was calculated, which corresponded to the point at which the sum of false positives and false negatives was less than at any other point. Sensitivity and specificity for selected cut-off points were then assessed. *P* values of < 0.05 were considered statistically significant.

## Results

### Clinical characteristics

The clinical characteristics of patients are shown in Table 1. There were differences in basal FSH concentration, AFC and AMH concentrations among the three groups, but no differences in age.

### Proteomics

At least two peptides were used for quantification and protein identification. For Protein Pilot-based database searching and identification, the threshold [unused protscore (conf)] was set to achieve 95% confidence at a 5% false discovery rate. The protein identification threshold, a ProtScore value > 1.3, was used to attain a confidence of 95%. When the proteins were classified as significantly regulated or not, an additional > 1.3 (1 × 1.3) or < 0.77 (1/1.3)-fold cutoff was applied to all iTRAQ ratios to minimize false positives when determining whether proteins were over-expressed or under-expressed. This cutoff value was used as overall technical variation of data from the duplicate experiments, and, in other investigations using the iTRAQ approach, this value has been widely used.

### Selection of target proteins

**Fig. 2:**
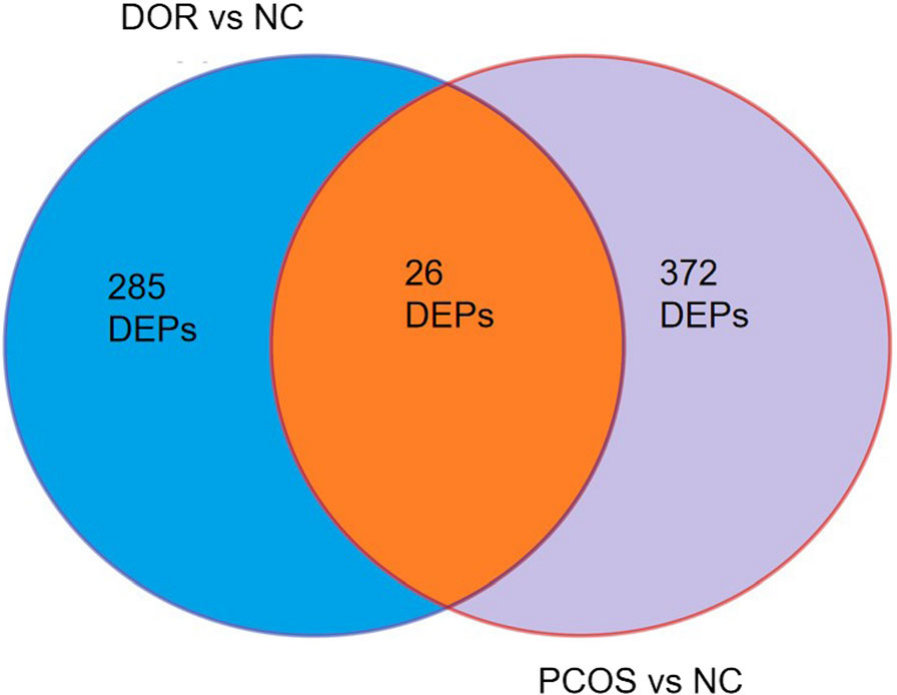
Differentially expressed urinary proteins (DEPs) in DOR and PCOS, compared with NC samples

**Fig. 3:**
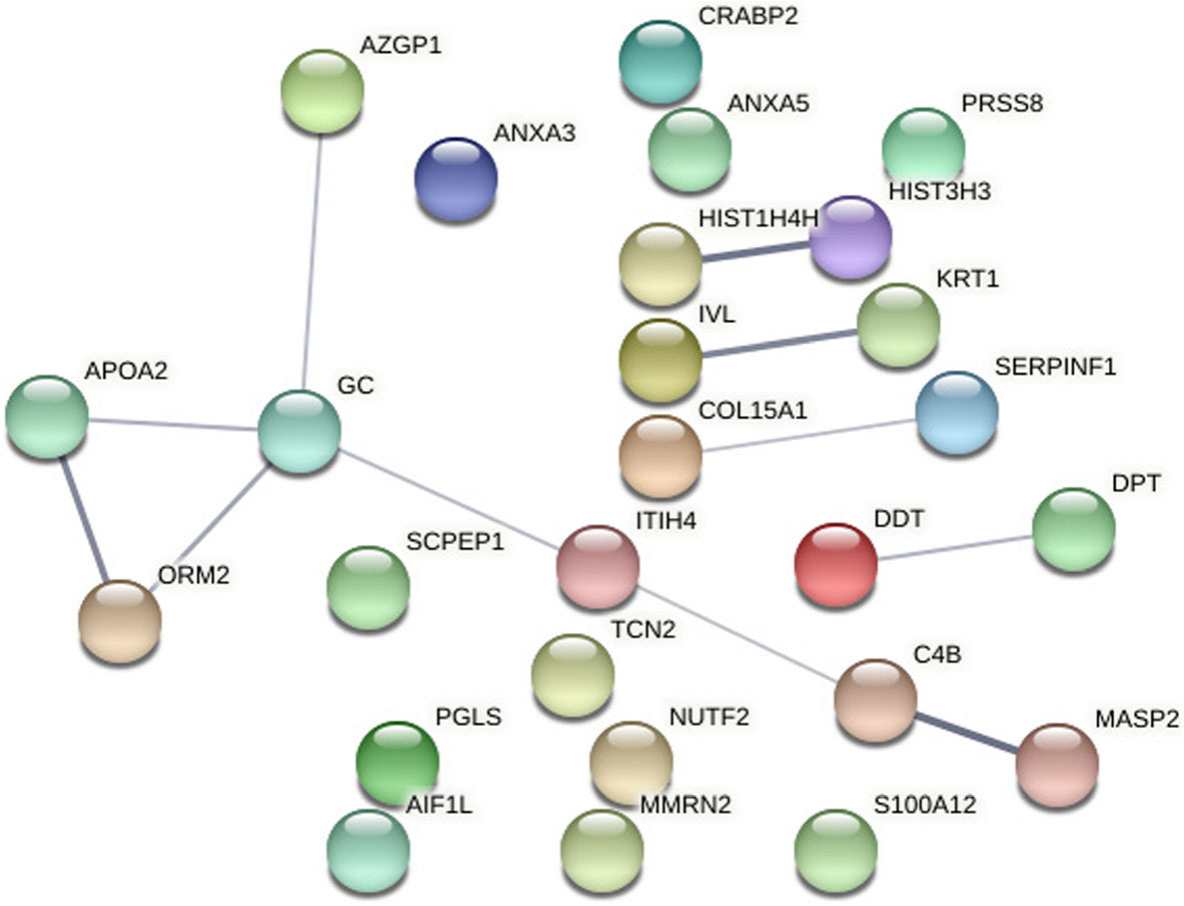
Protein-protein interaction (PPI) network (Vitamin D-binding protein is a protein encoded by the GC gene)

When compared with NC samples, 285 DEPs were identified in the DOR samples, and 372 DEPs in the PCOS samples. By analyzing the intersection of the two groups of DEPs, we found 26 proteins with different expression trends in the DOR and PCOS groups (Fig. 2 and Table 2). For example, a protein was under-expressed in DOR group and over-expressed in PCOS group, or the converse. To identify the key proteins among the 26 DEPs, we created the protein-protein interaction (PPI) network (Fig. 3) and identified VDBP is the key protein for the PPI.

**Table 2:**
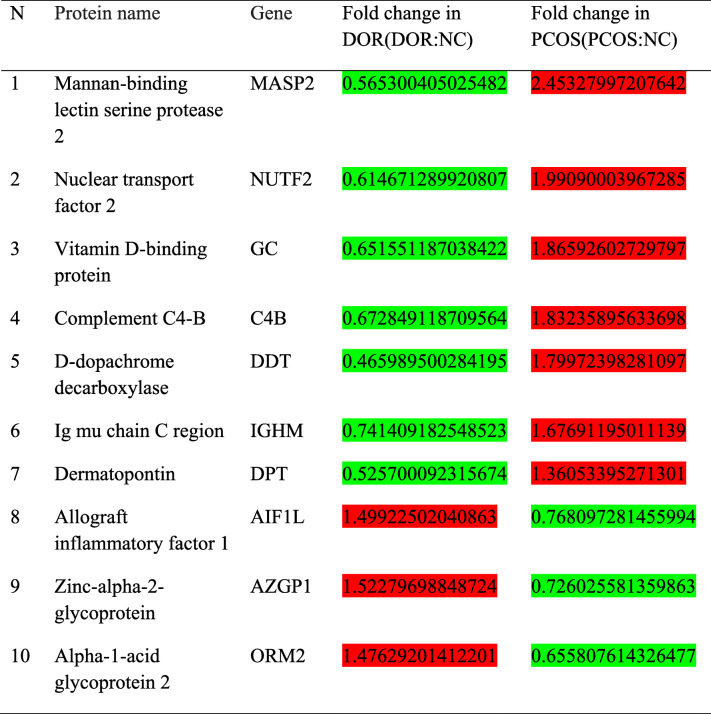
Partial list of differentially expressed urinary proteins in DOR and PCOS, compared with NC samples

Green is downregulated and red is upregulated

### Validation of VDBP

**Fig. 4:**
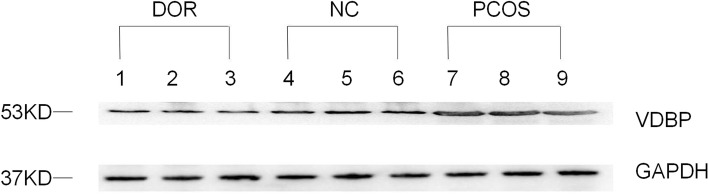
Western blot analyses of VDBP from DOR, PCOS and NC urine samples

**Fig. 5:**
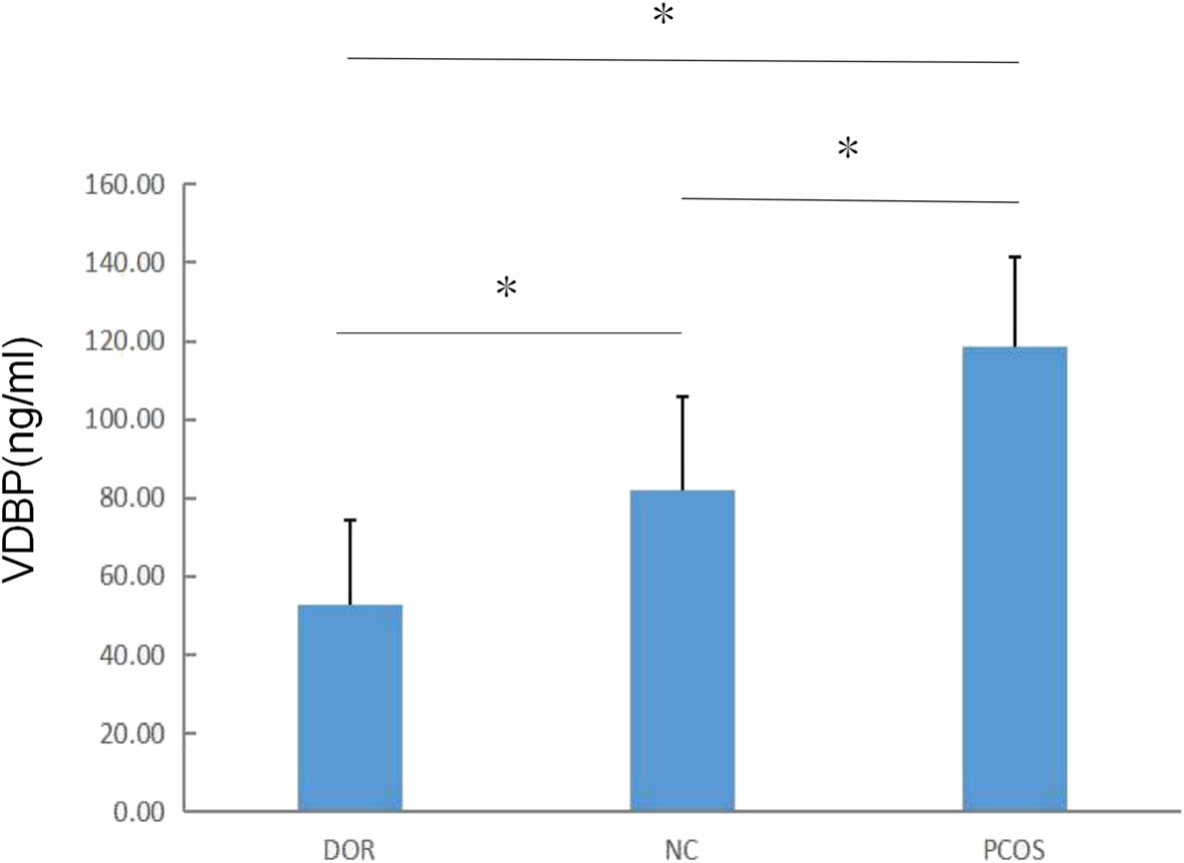
VDBP levels in the urine of patients in the DOR, PCOS and NC groups. Data are expressed as mean ± SD.*P < 0.05

**Fig. 6:**
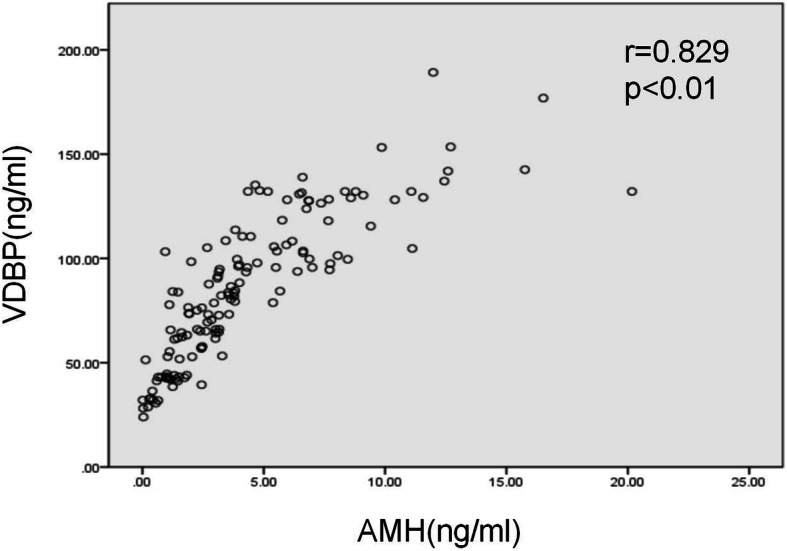
Scatter plot of linear relations between urinary VDBP level and serum AMH level

We validated the existence of VDBP by western blot analysis in the discovery cohort. VDBP was over-expressed in the urine of the PCOS group, and it was under-expressed in the DOR group (Fig. 4). Then we measured the levels of urinary VDBP in the validation cohort using ELISA, including patients with DOR (*n* = 43), NC (*n* = 49), and PCOS (*n* = 48). Comparison of urinary VDBP levels of the three groups of samples showed the highest levels in the PCOS group, median levels in the NC group, and the lowest levels in the DOR group (115.90 ± 26.02 ng/ml, 81.86 ± 23.92 and 52.84 ± 21.37, respectively; *P* < 0.05) (Fig. 5). Furthermore, urinary VDBP level was significantly and positively correlated with serum AMH level(*r* = 0.829, *P* < 0.01) (Fig. 6).

### Quantitative diagnostic analysis

ROC curves were used to assess the value of VDBP in evaluating ovarian reserve.

**Fig. 7:**
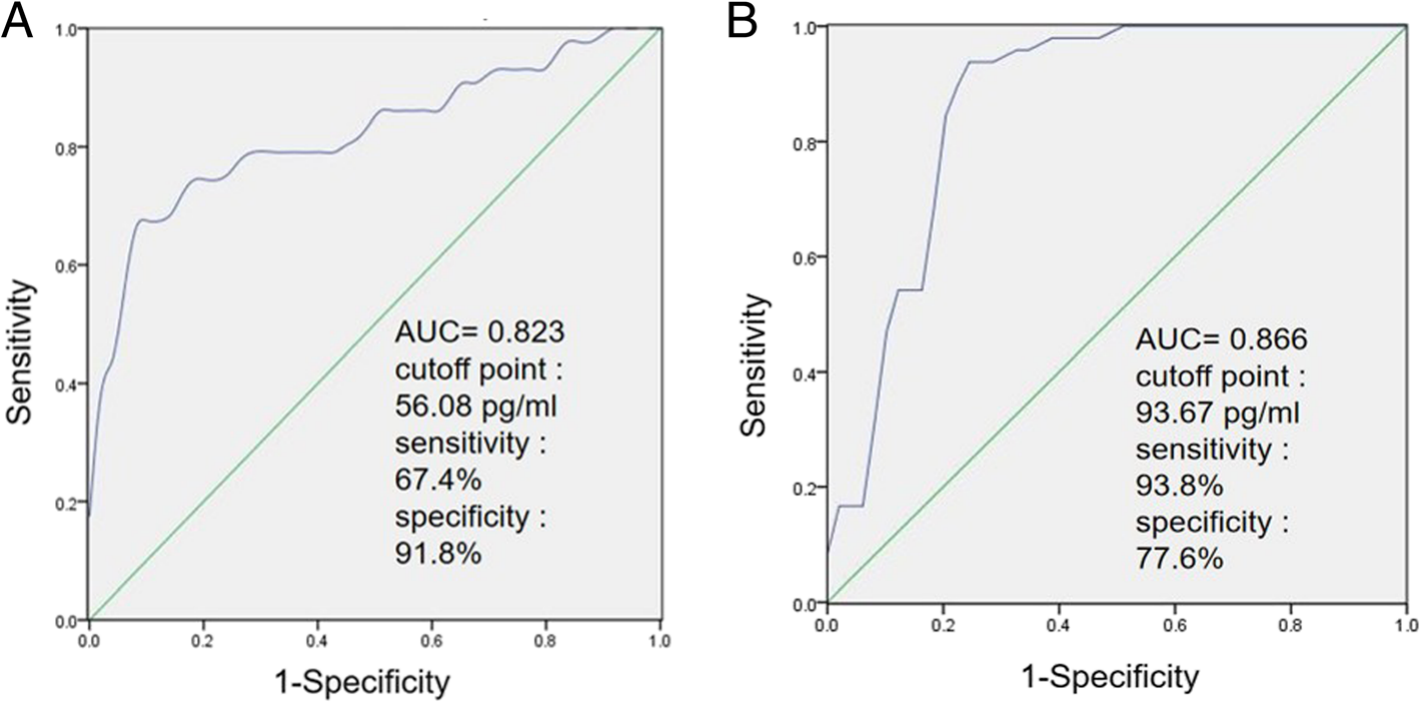
The receiver operating characteristic (ROC) analysis of urinary VDBP in the diagnosis of DOR (**A**) and PCOS (**B**)

The cutoff point was determined using the Youden index (Sensitivity + Specificity − 1) (35). When VDBP was used in the diagnosis of DOR, AUC of VDBP was 0.823 (95% CI: 0.734–0.912) with a sensitivity of 67.4% and a specificity of 91.8% when the cutoff point was set to 56.08 pg/ml (Fig. 7a). When VDBP was used in the diagnosis of PCOS, the area under the curve of VDBP was 0.866 (95% CI: 0.790–0.943) with a sensitivity of 93.8% and a specificity of 77.6% when the cutoff point was set to 93.67 pg/ml (Fig. 7b).

## Discussion

Blood and urine are two frequently media used for the discovery of biomarkers of human diseases as both can be sampled frequently and non-invasively. Urine as body fluid, however, has advantages over blood: 1) it can be easily obtained in large volumes and 2) the urinary proteome is less complex and has a relatively lower dynamic range allowing low abundance but functionally important proteins, such as exosomal proteins, to be reliably measured by LC-MS/MS [14–16].

Our study showed that urine VDBP was associated with ovarian reserve. Compared with the normal ovarian reserve group, VDBP expression was down-regulated in the DOR group and up-regulated in the PCOS group. Urinary VDBP level was significantly and positively correlated with serum AMH level- the most reliable marker of ovarian reserve to date.

Vitamin D-binding protein, originally known as a group-specific component (GC) of serum (GC-globulin), is a protein encoded by the GC gene. VDBP is a glycosylated alpha-globulin that is synthesized in the liver, although it can also be expressed in fat tissue, the kidneys, and gonads. It is composed of 458 amino acid residues in length and folds into a triple-domain structure bound by disulfide bonds. VDBP is the primary plasma carrier protein to which the metabolites of vitamin D are bound for transport around the body [17].

In the recent years, vitamin D has been in the spotlight in many fields of research, especially in human reproduction [18]. Vitamin D receptor expression was identified in ovarian granulosa cells as well as other female reproductive organs, including the endometrium and the uterus. These findings suggest that vitamin D may have a role in female reproduction [19–21]. Several studies have demonstrated a direct effect of vitamin D on ovarian folliculogenesis and steroidogenesis in animal and human cell-line studies. Vitamin D receptor null mutant mice have impaired folliculogenesis and vitamin D stimulated steroidogenesis in human ovarian cells [22, 23].

Some studies have suggested a significant association between vitamin D levels and ovarian reserve [24–26].Furthermore,a systematic review and meta-analysis of the interventional studies revealed that vitamin D supplementations affect serum AMH levels but have opposite effects depending on the ovulatory statuses of the women. It increased serum AMH levels in ovulatory non-PCOS women, while it decreased AMH levels in PCOS women [27]. On the contrary,most studies have not found any correlation between vitamin D and ovarian reserve markers [28–31].

The discrepancy might be explained by the free hormone hypothesis [32]. The free hormone hypothesis states that protein-bound hormones are relatively inactive, whereas hormones not bound to binding proteins are available to exert biologic activity. Total serum 25(OH) D is the most widely used biochemical marker to determine vitamin D status. Therefore, VDBP level can affect free 25(OH) D level. If an individual with low serum 25(OH)D, according to current parameters (less than 50 nM 25(OH) D, might nevertheless have adequate levels of free 25(OH)D if serum levels of VDBP are low or if the person has a GC genotype associated with a form of VDBP for which vitamin D metabolites exhibit a lower affinity. Even with the same total vitamin D levels, individuals with different ovarian reserve functions may have different levels of free vitamin D due to differences in VDBP.

In addition to vitamin metabolism, VDBP is also involved in the chemotaxis of other molecules, such as fatty acids and endotoxins, and has immunomodulatory properties. Importantly, VDBP is a component of the actin scavenger system that augments the pro-inflammatory response and clears the products of tissue injury, and it influences T cell responses and the VDBP-macrophage activating factor (DBP-MAF) that is involved in bone metabolism [33].

In recent years, many studies have shown that low grade chronic inflammation is associated with PCOS [34]. Inflammatory markers, including interleukin-18(IL-18), IL-6, tumor necrosis factor alpha (TNF-α) and C-reactive protein (CRP), are often elevated in patients with polycystic ovary syndrome, and may be early indicators of the risk of developing insulin resistance and cardiovascular system diseases, and may be useful prognostic and therapeutic tools for monitoring patients with PCOS [35–37]. Therefore, we hypothesized that the increased VDBP levels in the urine of PCOS patients were associated with chronic inflammation.

To the best of our knowledge, this study is the first to report on the identification and validation of VDBP as a urinary biomarker in the assessment of ovarian reserve. We found that as ovarian reserve function goes from “low” to “high”, urine VDBP levels were significantly increased. This study provides a novel way to find biomarkers of ovarian reserve. Although urine VDBP still has certain diagnostic limitations, it can be used as an new and non-invasive predictor for ovarian reserve. Studies involving large sample sets are needed to validate the results, and we look forward to seeing these results translated into clinical practice.

## Data Availability

The datasets used and analyzed during the current study are available from the corresponding author on reasonable request.

## Abbreviations

PCOS: Polycystic ovary syndrome
DOR: Diminished ovarian reserve
NC: Normal control
ELISA: Enzyme-linked immunosorbent assay
iTRAQ: isobaric Tags for Relative and Absolute Quantitation
DEPs: Differentially expressed proteins
VDBP: Vitamin D-binding protein
POR: Poor ovarian response
IVF: In vitro fertilization
FSH: Follicle-stimulating hormone
AMH: Anti-mullerian hormone
AFC: Antral follicle count
BMI: Body mass index
25(OH)D: 25-hydroxyvitamin D
LC-ESI-MS/MS: Liquid chromatography-electrospray ionization-tandem mass spectrometry
MCC: Maximal clique centrality
PPI: Protein-protein interaction
SNK: Student-Newman-Keuls
ROC: Receiver operating characteristic
AUC: Area under the ROC curve
GC: Group-specific component
DBP-MAF: VDBP-macrophage activating factor
IL: Interleukin
TNF-α: Tumor necrosis factor alpha
CRP: C-reactive protein
